# ^68^Ga-FAPI-04 Versus ^18^F-FDG PET/CT in the Detection of Hepatocellular Carcinoma

**DOI:** 10.3389/fonc.2021.693640

**Published:** 2021-06-25

**Authors:** Hao Wang, Wenwei Zhu, Shuhua Ren, Yanyan Kong, Qi Huang, Jun Zhao, Yihui Guan, Huliang Jia, Jinhong Chen, Lu Lu, Fang Xie, Lunxiu Qin

**Affiliations:** ^1^ Department of General Surgery, Huashan Hospital, Fudan University, Shanghai, China; ^2^ PET Centre, Huashan Hospital, Fudan University, Shanghai, China; ^3^ Department of Nuclear Medicine, Shanghai East Hospital, Tongji University School of Medicine, Shanghai, China

**Keywords:** ^68^Ga-FAPI-04, ^18^F-FDG, hepatocellular carcinoma (HCC), cancer-associated fibroblast (CAF), fibroblast activating protein (FAP)

## Abstract

**Background:**

Fibroblast activation protein (FAP) is commonly expressed in activated stromal fibroblasts in various epithelial tumours. Recently, ^68^Ga-FAPI-04 has been used for tumour imaging in positron emission tomography/computed tomography (PET/CT). This study aimed to compare the diagnostic performances of ^68^Ga-FAPI-04 PET/CT and ^18^F-FDG PET/CT in hepatocellular carcinoma (HCC), and to assess factors associated with ^68^Ga-FAPI-04 uptake in HCC.

**Materials and Methods:**

Twenty-nine patients with suspiciously HCC who received both ^18^F-FDG and ^68^Ga-FAPI-04 PET/CT were included in this retrospective study. The results were interpreted by two experienced nuclear medicine physicians independently. The maximum and mean standardized uptake values (SUV_max_ and SUV_mean_) were measured in the lesions and liver background, respectively. The tumour-to-background ratio (TBR) was then calculated as lesion’s SUV_max_ divided by background SUV_mean_.

**Results:**

A total of 35 intrahepatic lesions in 25 patients with HCC were finally involved in the statistical analysis. ^68^Ga-FAPI-04 PET/CT showed a higher sensitivity than ^18^F-FDG PET/CT in detecting intrahepatic HCC lesions (85.7% *vs*. 57.1%, *P* = 0.002), including in small (≤ 2 cm in diameter; 68.8% *vs*. 18.8%, *P* = 0.008) and well- or moderately-differentiated (83.3% *vs*. 33.3%, *P* = 0.031) tumors. SUV_max_ was comparable between ^68^Ga-FAPI-04 and ^18^F-FDG (6.96 ± 5.01 *vs*. 5.89 ± 3.38, *P* > 0.05), but the TBR was significantly higher in the ^68^Ga-FAPI-04 group compared with the ^18^F-FDG group (11.90 ± 8.35 *vs*. 3.14 ± 1.59, *P* < 0.001). SUV_max_ and the TBR in ^68^Ga-FAPI-04 positive lesions were associated with tumour size (both *P* < 0.05), but not the remaining clinical and pathological features (all *P* > 0.05).

**Conclusions:**

^68^Ga-FAPI-04 PET/CT is more sensitive than ^18^F-FDG PET/CT in detecting HCC lesions, and ^68^Ga-FAPI-04 uptake is correlated mainly with tumour size.

## Introduction

Hepatocellular carcinoma (HCC), the most frequent primary liver cancer, is the fourth most common cause of cancer-related death worldwide (1, 2). The majority of HCCs occur in patients with underlying liver disease, mostly as a result of hepatitis B or C virus (HBV or HCV) infection or alcohol abuse (3). Unlike many other malignant tumours, HCC can be diagnosed by imaging based on non-invasive criteria without confirmatory pathology (4). Therefore, imaging plays a critical role in the detection and diagnosis of HCC. Conventional imaging modalities, including computed tomography (CT), magnetic resonance imaging (MRI) and ultrasound, are mainly utilized for anatomical evaluation, with limited value in the assessment of morphologically atypical lesions (5). In contrast, positron emission tomography/computed tomography (PET/CT) as one of the functional imaging approaches has the potential to provide relevant biological information in HCC and to improve response assessment (6). Furthermore, a subset of HCCs cannot be diagnosed non-invasively either because the patients do not have cirrhosis or the lesions do not follow established enhancement patterns on contrast-enhanced CT or MRI (1, 3). Therefore, molecular imaging with PET/CT offers potential additional advantages to non-invasively confirm a diagnosis of HCC. However, the most widely available clinical PET tracer, ^18^F-FDG, shows poor sensitivity for the detection of HCC ranging from 40% to 68%, mainly because of the relatively high glucose-6-phosphatase activity found in low-grade HCC (6). In addition, it is not useful for the detection of small HCC lesions (7, 8). Therefore, several new tracers have been developed and applied for HCC detection, including ^11^C-acetate, ^11^C-choline, ^68^Ga-PSMA (9–12).

Cancer-associated fibroblasts (CAFs) are among the most crucial components of the tumour microenvironment that creates a favourable microenvironment for tumour growth, invasion and metastasis (13, 14). Fibroblast activation protein (FAP), a cell surface glycoprotein belonging to the serine protease family, is commonly expressed in activated stromal fibroblasts in various epithelial tumours (15, 16). Recent investigations indicated that ^68^Ga-labelled FAP inhibitor (FAPI) shows an equal or even improved tumour imaging with lower background uptake in the liver and the brain in comparison to ^18^F-FDG in various cancers (17, 18). ^68^Ga-FAPI-04 PET/CT was also revealed to have high sensitivity in detecting hepatic malignancies (19–21). Therefore, ^68^Ga-FAPI-04 may be a potential tracer for visualizing HCC by targeting CAFs that are abundant in the tumour microenvironment. This study aimed to comparatively assess the diagnostic performances of ^68^Ga-FAPI-04 PET/CT and ^18^F-FDG PET/CT in HCC and to assess factors associated with the uptake of ^68^Ga-FAPI-04 in HCC.

## Materials and Methods

### Patients

This is a post-hoc analysis of a prior prospective study conducted at the Huashan Hospital of Fudan University. Twenty-nine patients with suspiciously incipient or recurrent HCC determined by clinical manifestations and conventional imaging techniques (CT, MRI and ultrasound) were included in this retrospective study. They underwent both ^18^F-FDG and ^68^Ga-FAPI-04 PET/CT examinations with an interval of one day before surgical treatment. In patients who underwent surgery or biopsy, the definitive diagnosis was confirmed by pathology. In patients who underwent transarterial chemoembolization (TACE), HCC diagnosis was based on a specific imaging pattern of hyperenhancement in the arterial phase and washout in the venous or delayed phase, on contrast-enhanced CT or MRI in the setting of liver cirrhosis (1, 3). This study was approved by the institutional ethics committee, and written informed consent was obtained from all patients.

### PET/CT Imaging

Whole-body static FDG PET/CT scans were obtained as a routine procedure on a dedicated PET/CT scanner (Biograph mCT Flow scanner, Siemens, Germany). Whole-body ^68^Ga-FAPI-04 PET/CT scans were obtained on another PET/CT scanner (μMI510, Union imaging, Shanghai, China) within 60 min after intravenous injection of ~ 185 MBq (~5 mCi) of ^68^Ga-FAPI-04. Low-dose CT scans were obtained for attenuation correction and image fusion. PET images were acquired in the 3D mode, and reconstructed by the ordered subset expectation maximization 3D (OSEM 3D) method.

Because two different PETs were applied in this study, SUVs were normalized after data collection for PET/CT system performance harmonization. NEMA IEC body phantom (Data Spectrum Corporation, Durham, NC, USA) with 6 simulated lesion spheres (diameters of 10 mm, 13 mm, 17 mm, 22 mm, 28 mm and 37 mm, respectively) and 2, 4, 8, and 16 times of background activity (2 kBq/mL of background activity concentration) based on routine scan protocols was applied for SUV normalization.

### Image Evaluation

^18^F-FDG and ^68^Ga-FAPI-04 PET/CT images were interpreted independently by two experienced nuclear medicine physicians blinded to other imaging and pathology results. The maximum standardized uptake value (SUV_max_) was measured by delineating a spherical region of interest (ROI) for each lesion. The mean standardized uptake value (SUV_mean_) of the liver background was measured by drawing a spherical ROI with 2 cm diameter in the non-tumour hepatic parenchyma of the right lobe in each patient. The tumour-to-background ratio (TBR) was calculated by dividing the lesion’s SUV_max_ with the background SUV_mean_. A lesion was considered to be positive on the basis of the visual judgment of elevated uptake in the tumour tissue by 2 experienced nuclear medicine physicians independently, supported by the calculation of the TBRs of ^18^F-FDG and ^68^Ga-FAPI-04, respectively. Any difference of opinion between these two physicians was settled by mutual agreement.

### Statistical Analysis

All statistical analyses were conducted with the STATA statistical analysis software (StataCorp LLC, version 15.1). Categorical variables were presented as frequency and percentage, and continuous variables as mean ± standard deviation (SD). The McNemar’s test and Fisher exact test were performed to compare categorical variables. Non-parametric tests were carried out for the comparison of continuous variables with non-normal distribution. The Spearman rank correlation coefficient was determined to assess the correlation between continuous variables with non-normal distribution. Two-tailed *P* < 0.05 was considered statistically significant.

## Results

### Patient Characteristics

**Figure 1:**
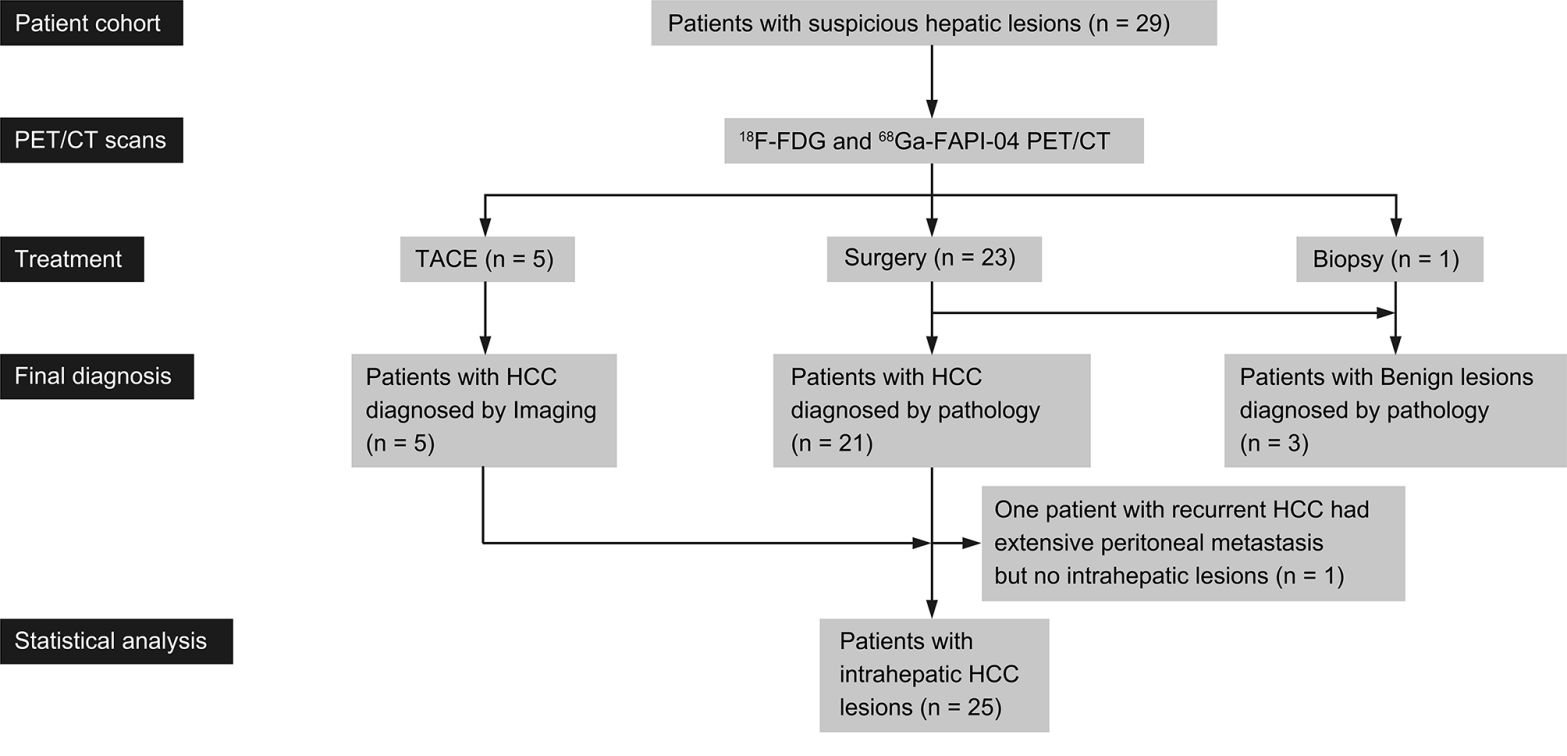
Study flowchart (n = number of patients).

Twenty-nine patients were included in the current study, including 23 treated by hepatic surgery, 5 administered TACE, and one that underwent biopsy only. Except for 3 patients who were diagnosed with benign hepatic nodules, the remaining 26 patients were diagnosed with HCC. One recurrent HCC case who underwent surgical resection had extensive peritoneal dissemination but no intrahepatic lesions. Therefore, 25 HCC patients with 35 intrahepatic lesions were finally involved in the statistical analysis. The study flowchart is presented in **Figure 1**. According to microvascular invasion (MVI) number and distribution, 2, 8 and 10 patients were categorized into the M0 (no MVI), M1 (≤ 5 MVI in adjacent liver tissue ≤ 1 cm away from the HCC), and M2 (> 5 MVI or MVI in adjacent liver tissue > 1 cm away from the HCC) groups, respectively. According to the American Joint Committee on Cancer (AJCC) cancer staging system (8th Edition), 5, 10, 4 and 1 patients were categorized into stage I, II;, III and IV, respectively. The general characteristics of the 25 HCC patients are summarized in **Table 1**.

**Table 1:** Characteristics of the included HCC patients.

General characteristics	n = 25	%
Age (years)	59.40 ± 6.90	
Gender (male)	24	96
HBsAg (+)	20	80
Anti-HCV (+)	0	0
Cirrhosis	19	76
AFP (> 20 ng/mL)	12	48
Tumour number		
Solitary tumour	15	60
Multiple tumours	10	40
MVI		
M0	2	8
M1	8	32
M2	10	40
AJCC TNM stage		
I	5	20
II	10	40
III	4	16
IV	1	4

Five HCC patients whose diagnosis was based on non-invasive criteria underwent TACE instead of hepatic surgery and, therefore, had no pathological data. HBsAg, hepatitis B surface antigen; Anti-HCV, anti-hepatitis C virus antibody; AFP, α-fetoprotein; MVI, microvascular invasion; AJCC TNM, American Joint Committee on Cancer tumour-node-metastasis.

### Comparison of ^68^Ga-FAPI-04 With ^18^F-FDG in Patient-Based Analysis

**Figure 2:**
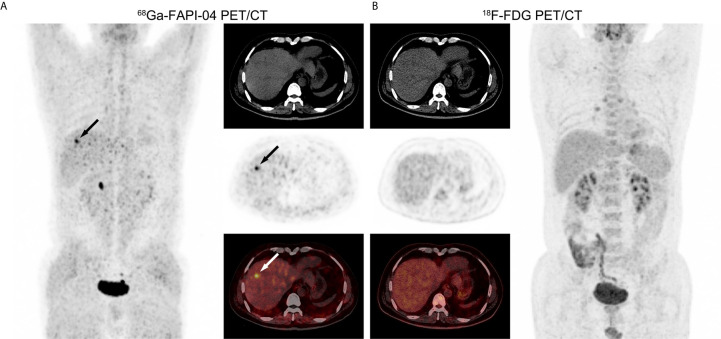
PET/CT images in a 53-year-old male patient with moderately-differentiated HCC. **(A)** ^68^Ga-FAPI-04 PET/CT revealed a strongly FAPI-avid lesion (black and white arrows, SUV_max_ = 7.36, TBR = 6.03) in the right lobe of the liver. **(B)** No positive finding was observed within liver in ^18^F-FDG PET/CT images (SUV_max_ = 2.36, TBR = 1.31).

The results of the patient-based analysis of ^18^F-FDG and ^68^Ga-FAPI-04 PET/CT are summarized in **Table 2**. Of the 35 intrahepatic HCCs, 20 were tested positive by both ^18^F-FDG and ^68^Ga-FAPI-04 PET/CT, 10 were tested positive by ^68^Ga-FAPI-04 PET/CT alone, and 5 were not tested positive by either method. ^68^Ga-FAPI-04 PET/CT showed a better sensitivity in detecting intrahepatic lesions compared with ^18^F-FDG PET/CT (85.7% *vs*. 57.1%, *P* = 0.002). In subgroup analysis, ^68^Ga-FAPI-04 PET/CT was more sensitive than ^18^F-FDG PET/CT in the detection of intrahepatic lesions in patients with cirrhosis, low α-fetoprotein (AFP), multiple tumours, and non-serious MVI (M0 and M1) (all *P* < 0.05). Moreover, ^68^Ga-FAPI-04 PET/CT detected 4 of the 5 lesions in patients with stage I disease, whereas ^18^F-FDG PET/CT did not reveal any abnormal finding in these patients (**Figure 2**). The sensitivity of ^18^F-FDG PET/CT was associated with AJCC TNM stage (*P* = 0.016), while that of ^68^Ga-FAPI-04 PET/CT was correlated with serum AFP levels (*P* = 0.045). These findings suggested that ^68^Ga-FAPI-04 PET/CT was more sensitive than ^18^F-FDG PET/CT in the detection of intrahepatic lesions, particularly in patients with cirrhosis, low AFP, multiple HCCs, and non-serious MVI.

**Table 2:** Sensitivities of ^18^F-FDG and ^68^Ga-FAPI-04 PET/CT in patient-based analysis.

Patient characteristics	No. of patients	No. oflesions	^18^F-FDG		^68^Ga-FAPI-04		*P* between 2 tracers
			Positive lesions (%)	*P*	Positive lesions (%)	*P*	
All	25	35	20 (57.1)		30 (85.7)		0.002^*^
Clinical features							
Cirrhosis	19	29	16 (55.2)	0.680	24 (82.8)	0.561	0.008^*^
Non-cirrhosis	6	6	4 (66.7)		6 (100)		0.500
AFP (ng/mL)							
≤ 20	13	17	11 (64.7)	0.500	17 (100)	0.045^*^	0.031^*^
> 20	12	18	9 (50.0)		13 (72.2)		0.125
Tumour number							
Solitary tumour	15	15	10 (66.7)	0.492	14 (93.3)	0.365	0.125
Multiple tumours	10	20	10 (50.0)		16 (80.0)		0.031^*^
MVI							
M0 + M1	10	14	4 (28.6)	0.057	10 (71.4)	0.326	0.031^*^
M2	10	14	10 (71.4)		13 (92.9)		0.250
AJCC TNM staging							
I	5	5	0 (0)	0.016^*^	4 (80.0)	0.423	0.125
II	10	16	8 (50.0)		12 (75.0)		0.125
III + IV	5	7	6 (85.7)		7 (100)		1

Five HCC patients whose diagnosis was based on non-invasive criteria underwent TACE instead of hepatic surgery and, therefore, had no pathological data. ^*^, statistically significant; AFP, α-fetoprotein; MVI, microvascular invasio; AJCC TNM, American Joint Committee on Cancer tumour-node-metastasis.

### Comparison of ^68^Ga-FAPI-04 With ^18^F-FDG in Lesion-Based Analysis

The results of lesion-based analysis of ^18^F-FDG and ^68^Ga-FAPI-04 PET/CT are summarized in **Table 3**. ^68^Ga-FAPI-04 PET/CT was more sensitive than ^18^F-FDG PET/CT in detecting small HCCs (≤ 2 cm in diameter) (*P* = 0.008) and well- or moderately-differentiated HCCs (*P* = 0.031), but there were no significant sensitivity differences between the 2 tracers in the detection of HCCs > 2 cm in diameter (both *P* > 0.05) and poorly-differentiated or undifferentiated HCCs (*P* > 0.05). The sensitivities of ^18^F-FDG and ^68^Ga-FAPI-04 PET/CT were significantly related to the size of intrahepatic lesions (both *P* < 0.05). These findings indicated that ^68^Ga-FAPI-04 PET/CT was more sensitive than ^18^F-FDG PET/CT in the detection of small and well- or moderately-differentiated HCCs.

**Table 3:** Sensitivities of ^18^F-FDG and ^68^Ga-FAPI-04 PET/CT in lesion-based analysis.

Lesion characteristics	No. of lesions	^18^F-FDG		^68^Ga-FAPI-04		*P* between 2 tracers
		Positive lesions (%)	*P*	Positive lesions (%)	*P*	
Diameter (cm)						
≤ 2	16	3 (18.8)	<0.001^*^	11 (68.8)	0.038^*^	0.008^*^
> 2, ≤ 5	11	9 (81.8)		11 (100)		0.500
> 5	8	8 (100)		8 (100)		1
Histologic grade						
I + II	12	4 (33.3)	0.252	10 (83.3)	1	0.031^*^
III + IV	15	9 (60.0)		12 (80.0)		0.250

Eight lesions had no pathological data.

^*^Statistically significant.

### Uptake Intensities of ^18^F-FDG and ^68^Ga-FAPI-04 in HCC

**Figure 3:**
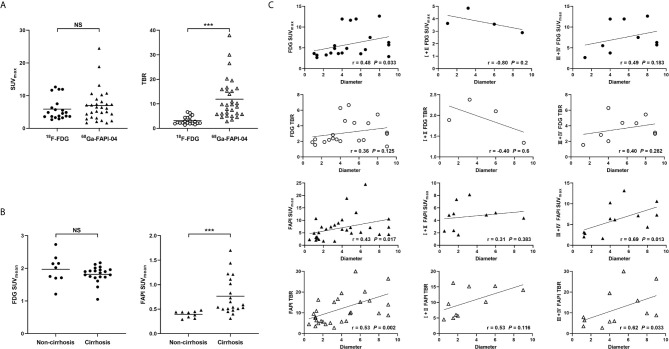
**(A)** Comparison of uptake intensity (SUV_max_ and TBR) between ^18^F-FDG and ^68^Ga-FAPI-04. **(B)** Comparison of background uptake (SUV_mean_) between patients with cirrhosis or those without cirrhosis. **(C)** Spearman rank correlation analysis of uptake intensity (SUV_max_ and TBR) and tumour diameter in ^18^F-FDG and ^68^Ga-FAPI-04. NS, not statistically significant; ***, P < 0.001.

Among the 25 HCC patients with 35 intrahepatic lesions, uptake of ^18^F-FDG and ^68^Ga-FAPI-04 in positive lesions was assessed, respectively (**Table 4**). Although the lesion uptake (SUV_max_) of ^68^Ga-FAPI-04 was similar to that of ^18^F-FDG (*P* > 0.05), its TBR was significantly higher than that of ^18^F-FDG (*P* < 0.001) (**Figure 3A**). Particularly, the background uptake (SUV_mean_) of ^68^Ga-FAPI-04 was much lower than that of ^18^F-FDG in each patient. The SUV_mean_ of ^68^Ga-FAPI-04 in patients with cirrhosis was significantly higher than that of patients without cirrhosis (0.76 ± 0.39 *vs*. 0.40 ± 0.07, *P* < 0.001); however, no significant difference was obtained in SUV_mean_ of ^18^F-FDG between these two groups (1.82 ± 0.39 *vs*. 1.97 ± 0.44, *P* > 0.05) (**Figure 3B**).

**Table 4:** Uptake intensities of ^18^F-FDG and ^68^Ga-FAPI-04 in positive lesions.

Characteristic	^18^F-FDG					^68^Ga-FAPI-04				
	No.	SUV_max_	*P*	TBR	*P*	No.	SUV_max_	*P*	TBR	*P*
All	20	5.89 ± 3.38		3.14 ± 1.59		30	6.96 ± 5.01		11.90 ± 8.35	
Clinical features										
Cirrhosis	16	5.43 ± 2.79	0.706	3.09 ± 1.57	0.925	24	7.29 ± 5.27	0.351	11.33 ± 8.41	0.300
Non-cirrhosis	4	7.77 ± 5.25		3.32 ± 1.92		6	5.61 ± 3.91		14.15 ± 8.47	
AFP (ng/mL)										
≤ 20	11	4.92 ± 2.55	0.239	2.60 ± 1.32	0.119	17	7.02 ± 5.27	0.818	10.72 ± 6.33	0.517
> 20	9	7.09 ± 4.01		3.79 ± 1.73		13	6.88 ± 4.86		13.44 ± 10.52	
Tumour number										
Solitary tumour	10	7.15 ± 4.26	0.326	3.73 ± 1.98	0.326	14	8.53 ± 6.43	0.271	14.69 ± 9.73	0.074
Multiple tumours	10	4.64 ± 1.58		2.54 ± 0.81		16	5.58 ± 2.89		9.45 ± 6.26	
MVI										
M0 + M1	4	3.64 ± 0.86	0.048^*^	2.02 ± 0.47	0.120	10	4.86 ± 2.04	0.852	10.45 ± 4.52	0.804
M2	10	7.16 ± 3.74		3.48 ± 1.58		13	5.85 ± 3.69		11.60 ± 8.78	
AJCC TNM staging										
I	0	–	0.093	–	0.121	4	4.07 ± 2.59	0.404	6.73 ± 2.36	0.194
II	8	4.92 ± 2.96		2.64 ± 1.53		12	4.99 ± 2.54		10.27 ± 5.70	
III + IV	6	7.79 ± 3.85		3.63 ± 1.37		7	6.94 ± 3.87		15.03 ± 9.56	
Diameter (cm)										
≤ 2	3	3.17 ± 0.49	0.079	1.90 ± 0.36	0.215	11	4.17 ± 2.75	0.023^*^	7.56 ± 3.62	0.019^*^
> 2, ≤ 5	9	5.91 ± 3.42		3.46 ± 1.89		11	7.84 ± 4.28		12.12 ± 10.15	
> 5	8	6.89 ± 3.65		3.23 ± 1.39		8	9.58 ± 6.77		17.55 ± 7.56	
Histologic grade										
I + II	4	3.74 ± 0.82	0.045^*^	1.93 ± 0.44	0.045^*^	10	4.62 ± 2.10	0.391	10.17 ± 4.70	0.895
III + IV	9	7.55 ± 3.74		3.66 ± 1.57		12	6.26 ± 3.67		11.90 ± 9.05	

No., number of positive lesions. ^*^Statistically significant; AFP, α-fetoprotein; MVI, microvascular invasion; AJCC TNM, American Joint Committee on Cancer tumour-node-metastasis.

The SUV_max_ of ^18^F-FDG in positive lesions was associated with the degree of MVI (*P* = 0.048) and tumour differentiation (*P* = 0.045), while the TBR was only associated with tumour differentiation (*P* = 0.045). In contrast, the SUV_max_ and TBR of ^68^Ga-FAPI-04 in positive lesions were associated only with tumour size (both *P* < 0.05), but not with other clinical and pathological features (all *P* > 0.05). In Spearman correlation analysis (**Figure 3C**), the SUV_max_ and TBR of ^68^Ga-FAPI-04 in positive lesions were correlated with tumour size (rSUV_max_ = 0.43, rTBR = 0.53, both *P* < 0.05). Further subgroup analysis revealed these correlations in poorly-differentiated or undifferentiated HCCs (rSUV_max_ = 0.69, rTBR = 0.62, both *P* < 0.05), rather than well- or moderately-differentiated ones (both *P* > 0.05). For ^18^F-FDG, SUV_max_, but not TBR, was correlated with tumour size (rSUV_max_ = 0.48, *P* = 0.033). However, neither SUV_max_ nor TBR exhibited a correlation with tumour size in subgroup analysis (all *P* > 0.05).

### Characteristics of Extrahepatic Metastases and Other Benign Lesions

**Figure 4:**
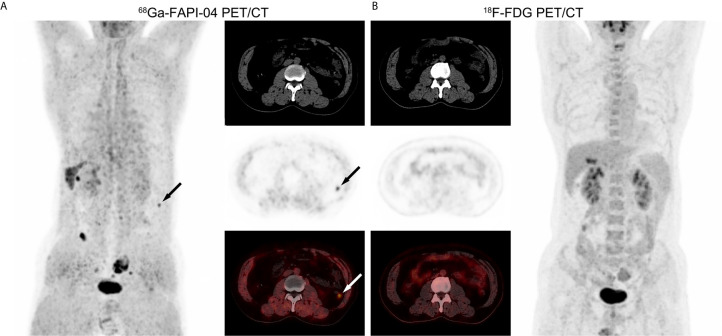
PET/CT images in a 51-year-old male patient with poorly-differentiated HCC and regional lymph node metastasis. **(A)** ^68^Ga-FAPI-04 PET/CT revealed a strongly FAPI-avid lesion (black and white arrows, SUV_max_ = 6.67, TBR = 15.2) that was pathologically confirmed as lymph node metastasis of the porta hepatis. **(B)** ^18^F-FDG PET/CT showed no elevated uptake of this extrahepatic metastasis (SUV_max_ = 2.84, TBR = 1.04).

**Figure 5:**
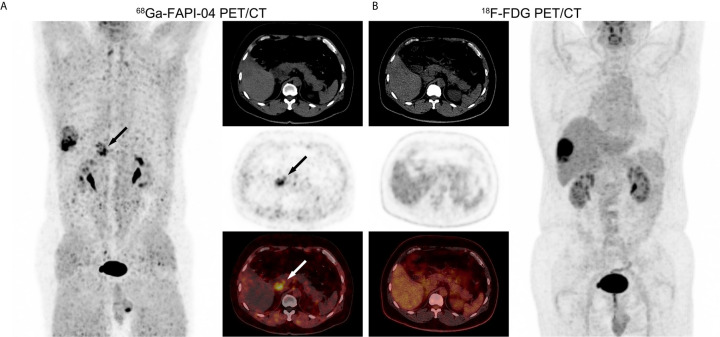
PET/CT images in a 53-year-old male patient with recurrent HCC and extensive peritoneal dissemination. **(A)** In ^68^Ga-FAPI-04 PET/CT, a small metastatic lesion confirmed by pathology showed elevated uptake (black and white arrows, SUV_max_ = 4.72, TBR = 11). **(B)** ^18^F-FDG PET/CT did not detect this metastatic lesion (SUV_max_ = 1.27, TBR = 0.63).

Lymph node metastasis in one patient with poorly-differentiated HCC showed strong uptake of ^68^Ga-FAPI-04, but undetectable uptake of ^18^F-FDG (**Figure 4**). ^68^Ga-FAPI-04 PET/CT detected a small metastatic lesion that was not revealed by ^18^F-FDG PET/CT in another HCC patient with extensive peritoneal dissemination (**Figure 5**).

Of the benign lesions, angiomyolipoma (AML) presented strong uptake of ^68^Ga-FAPI-04 (SUV_max_ = 8.34 and TBR = 21.92) and mildly increased uptake of ^18^F-FDG (SUV_max_ = 2.70 and TBR = 1.54), whereas focal nodular hyperplasia (FNH) presented elevated uptake of ^68^Ga-FAPI-04 (SUV_max_ = 2.20 and TBR = 5.77) and negative uptake of ^18^F-FDG (SUV_max_ = 1.51 and TBR = 0.86). In two cases of inflammatory nodules in the liver, one showed positive uptake of ^68^Ga-FAPI-04 (SUV_max_ = 1.56 and TBR = 3.08) and negative uptake of ^18^F-FDG (SUV_max_ = 2.14 and TBR = 1.19), while the other was neither ^68^Ga-FAPI-04 avid (SUV_max_ = 0.53 and TBR = 1.20) nor ^18^F-FDG avid (SUV_max_ = 2.92 and TBR = 1.36).

## Discussion

Nowadays, ^18^F-FDG as the most widely available clinical PET tracer has been increasingly utilized for detecting extrahepatic metastases (22), TNM staging (23), selecting patients for liver transplantation (23), and predicting tumour progression or recurrence after treatments (24, 25). However, ^18^F-FDG has limited value in the early diagnosis of HCC because of its low sensitivity (6, 26). Therefore, there remains an urgent need for highly sensitive tracers in the early diagnosis of HCC by PET/CT. Recently, ^68^Ga-labelled FAPI was shown to be a novel tracer in PET/CT imaging of various cancers due to its high tumour-to-background contrast (17, 27). Furthermore, ^68^Ga-FAPI-04 PET/CT has high sensitivity in detecting hepatic malignancies, including HCC and ICC (19, 21).

In line with previous studies reporting a range from 40% to 68%, the sensitivity of ^18^F-FDG PET/CT in the detection of HCC was 57.1% in the present study (6). ^68^Ga-FAPI-04 PET/CT had a better sensitivity (85.7%) than ^18^F-FDG PET/CT in the detection of intrahepatic lesions in HCC patients. Of note, ^68^Ga-FAPI-04 PET/CT was capable of detecting more than half of small HCC lesions (11 of 16, ≤ 2 cm in diameter) in the present cohort, whereas ^18^F-FDG PET/CT detected only three of the 16 lesions, which is consistent with previous studies that consider ^18^F-FDG an inappropriate tracer for visualizing small HCCs (7, 8). Furthermore, ^68^Ga-FAPI-04 PET/CT exhibited a relatively higher sensitivity in the detection of well- or moderately-differentiated HCCs (10 of 12, histologic grade I or II) compared with ^18^F-FDG PET/CT (4 of 12). The poor sensitivity of ^18^F-FDG PET/CT in detecting low-grade HCC is probably related to enhanced glucose-6-phosphatase activity causing the dephosphorylation of ^18^F-FDG-6-PO_4_, which is therefore not trapped in HCC cells, resulting in false-negative results (6, 28, 29).

In contrast, a CAF-targeting tracer can circumvent highly heterogeneous avidity exhibited by some tracers that target the tumour per se, because CAFs are among the most abundant stromal components in the tumour microenvironment of many solid tumours, and are found even at the early stages of tumorigenesis (30, 31). The above correlation analysis of ^68^Ga-FAPI-04 revealed that neither positive incidence nor uptake intensity was associated with tumour differentiation. Moreover, ^68^Ga-FAPI-04 showed a high lesion-to-background contrast in the liver, which may partially increase sensitivity that is affected by the partial volume effect of PET/CT. As such, ^68^Ga-FAPI-04 PET/CT can make up for the deficiencies of ^18^F-FDG PET/CT in the detection of low-grade HCC. Additionally, a higher detection rate was observed with ^68^Ga-FAPI-04 PET/CT in high-grade HCC in comparison with ^18^F-FDG PET/CT, although this difference was not statistically significant. Therefore, ^68^Ga-FAPI-04 PET/CT appears to be a promising new approach for the detection of intrahepatic HCC lesions with higher sensitivity compared with ^18^F-FDG PET/CT.

^68^Ga-FAPI-04 PET/CT could not detect 5 intrahepatic HCC lesions with the diameter within 2 cm in this cohort. These negative results may be due to the similar uptake intensity of ^68^Ga-FAPI-04 between small HCC lesions and the liver background of cirrhosis. Hypoxia may be a reasonable explanation for the positive correlation between the uptake intensity of ^68^Ga-FAPI-04 and tumour size. It has been reported that the degree of hypoxia correlates positively with tumour size (32) and that hypoxia is a potent factor inducing the expression of FAP in CAFs (33). Therefore, the degree of hypoxia is mild in small HCCs, leading to the low uptake of ^68^GA-FAPI-04 in these lesions. In the cirrhotic liver, FAP is strongly expressed by activated hepatic stellate cells (34, 35). In line with a previous study (19), this work also found that patients with cirrhosis presented elevated uptake of ^68^Ga-FAPI-04 in the hepatic parenchyma compared with those without cirrhosis. Therefore, small lesions have relatively lower uptake of ^68^Ga-FAPI-04 in comparison with large ones, which makes them susceptible to being masked by the background of cirrhosis.

Although ^18^F-FDG PET/CT has a potential value in detecting extrahepatic metastases in HCC patients (7, 8, 36, 37), heterogeneous uptake of ^18^F-FDG in metastatic nodules remains a major reason limiting its wide application for tumour staging. CAFs play a critical role in constructing a microenvironment that favours tumour progression at the primary site, and are, moreover, responsible for creating a pre-metastatic niche in distal organs and triggering the subsequent metastatic events (30, 31, 38). Therefore, CAF-targeting tracers seem to be optimal candidates for PET/CT in the evaluation of extrahepatic metastases. In the present study, lymph node metastasis at the porta hepatis in one patient with poorly-differentiated HCC presented a clear visualization in ^68^Ga-FAPI-04 PET/CT, but an obscure image in ^18^F-FDG PET/CT (**Figure 4**). Separately, one small metastatic lesion in another recurrent HCC case with extensive peritoneal dissemination was only clearly visualised by ^68^Ga-FAPI-04 PET/CT (**Figure 5**). It appears that ^68^Ga-FAPI-04 PET/CT may outperform ^18^F-FDG PET/CT in detecting extrahepatic lesions in patients with advanced HCC. Nevertheless, the comparison of ^68^Ga-FAPI-04 with ^18^F-FDG in terms of applicability to the detection of extrahepatic metastasis of HCC needs to be clarified in future studies.

Despite the high sensitivity of ^68^Ga-FAPI-04 PET/CT in the detection of malignancies, some benign lesions confirmed by pathological examinations in the current cohort presented positive results as well. Especially, AML, FNH, and one of two inflammatory nodules presented elevated uptake of ^68^Ga-FAPI-04 in contrast to the hepatic background, perhaps because of the enhanced fibrosis around or within lesions. Moreover, increasing uptake of ^68^Ga-FAPI-04 was observed in the postoperative area of the liver in one patient with recurrent HCC, which is consistent with a previous study that considered ^68^Ga-FAPI-04 an inappropriate tracer for the discrimination between abnormal malignant progression and normal postoperative reaction (39). Nevertheless, negligible ^68^Ga-FAPI-04 uptake has been observed in some other benign hepatic lesions such as adenoma (20, 39), dysplastic nodule (20), granuloma (21), and haemangioma (21). Collectively, great caution should be exercised when regarding intrahepatic lesions with elevated uptake of ^68^Ga-FAPI-04 as malignancy.

There were several limitations in the present study. First, it is unethical to biopsy all lesions as part of a research study, although that is perhaps not practical or needed. As a result, the lack of pathological data in 5 HCC patients whose confirmative diagnosis was based on non-invasive criteria may yield latent bias. Another limitation is that the present cohort only included a small number of patients with suspicious HCC who were willing to receive both ^68^Ga-FAPI-04 and ^18^F-FDG PET/CT examinations. Therefore, selection bias was inevitable. Finally, different scanners were used in this study for ^68^Ga-FAPI-04 and ^18^F-FDG imaging because of the different production places of PET tracers. For this reason, SUV normalization was applied after data collection for PET/CT system performance harmonization.

## Conclusions

^68^Ga-FAPI-04 PET/CT is more sensitive than ^18^F-FDG PET/CT in detecting intrahepatic HCCs. This outperformance is more prominent in the detection of small and well- or moderately-differentiated HCCs. The uptake of ^68^Ga-FAPI-04 was correlated mainly with tumour size in this study; therefore, ^68^Ga-FAPI-04 PET/CT can be considered a very promising imaging modality in HCC diagnosis.

## Data Availability Statement

The raw data supporting the conclusions of this article will be made available by the authors, without undue reservation.

## Ethics Statement

The studies involving human participants were reviewed and approved by ethics committee at Huashan Hospital of Fudan University. The patients/participants provided their written informed consent to participate in this study. Written informed consent was obtained from the individual(s) for the publication of any potentially identifiable images or data included in this article.

## Author Contributions 

LQ, FX, LL, WZ, and HW designed the study, interpreted the data and led the writing and review of the manuscript. HW, HJ, JC, WZ, and LL enrolled patients and collected clinical data. YG, JZ, QH, YK, and SR performed examinations. All authors contributed to the article and approved the submitted version.

## Funding

This study was funded by the Key Program of National Natural Science Foundation of China (81930074), the Major Program of National Natural Science Foundation of China (91959203), the Shanghai Sailing Program (18YF1403200), the Start-up fund of Huashan Hospital, Fudan University (2017QD081), and the Shanghai Municipal Key Clinical Specialty (shslczdzk03402).

## Conflict of Interest

The authors declare that the research was conducted in the absence of any commercial or financial relationships that could be construed as a potential conflict of interest.
